# Associations of Cumulative Vascular Risk Factors With Cerebral Perfusion

**DOI:** 10.1093/geroni/igaf122.2269

**Published:** 2025-12-31

**Authors:** Nazm Rahat, Chad Blackshear, A J Spychalla, Farwa Ali, Kevin Sullivan, Michael Griswold, Prashanthi Vemuri, B Gwen Windham

**Affiliations:** University of Mississippi Medical Center, Jackson, Mississippi, United States; University of Mississippi Medical Center - The MIND Center, Jackson, Mississippi, United States; Mayo Clinic, Rochester, Minnesota, United States; Mayo Clinic, Rochester, Minnesota, United States; University of Mississippi Medical Center - The MIND Center, Jackson, Mississippi, United States; University of Mississippi Medical Center - The MIND Center, Jackson, Mississippi, United States; Mayo Clinic, Rochester, Minnesota, United States; University of Mississippi Medical Center - The MIND Center, Jackson, Mississippi, United States

## Abstract

Lower cerebral perfusion is associated with adverse outcomes, but risk factors are poorly understood. This study examined associations of cumulative systolic blood pressure [SBP], diabetes, and BMI from mid- to late-life with late-life cerebral perfusion, using whole-brain MRI Arterial Spin Labeling (ASL). Cumulative exposures were defined as either average continuous SBP or BMI across visits or number of years with diabetes, hypertension, or obesity. Multiple regression, adjusted for age and gender, estimated ASL (β: 95% CI) among participants without prevalent conditions at baseline in participants with ASL data from the Atherosclerosis Risk in Communities Study (ARIC; Jackson, MS; n = 100; age=81±3 years; 65% Female; 100% Black participants; 34 years retrospective risk factor assessments (1987-2021); pulsed ASL, 8/2021-1/2024) and the Mayo Clinic Study of Aging (MCSA; Rochester, MN; n = 537; age=71±13 years; 36.3% Female, 100% White participants; 18 years retrospective risk factor assessments (2005-2023); pseudo-continuous ASL 2/2023-3/2024). A 10 mmHg higher cumulative SBP was associated with a 0.04 lower ASL (MCSA β=-0.04; -0.01, -0.07; ARIC β=-0.04; -0.09, 0.01). A 10 kg/m2 higher cumulative BMI was associated with a 0.30 lower ASL (-0.51, -0.08) in ARIC and 0.03 lower ASL (-0.16, 0.10) in MCSA participants. Every five additional years of diabetes exposure was associated with a 0.03 (-0.06, -0.01) lower ASL in ARIC and 0.11 (-0.27, 0.05) lower ASL in MCSA participants. High BP, obesity, and diabetes may contribute to poor cerebral perfusion over extended exposures from midlife. Longer risk factor assessment periods and different ASL techniques may explain differences in the cohorts.

